# A Security-Enhanced Certificateless Aggregate Signature-Based Conditional Privacy-Preserving Authentication Scheme for VANETs

**DOI:** 10.3390/s26175603

**Published:** 2026-09-03

**Authors:** Ruimin Wang, Can Liu, Hanbing Zhang, Mengyu Jia

**Affiliations:** 1 School of Computer and Artificial Intelligence, Zhengzhou University, Zhengzhou 450001, China; z202412842028348@gs.zzu.edu.cn (C.L.); zhb1126@gs.zzu.edu.cn (H.Z.); 2 School of Cyber Science and Engineering, Zhengzhou University, Zhengzhou 450002, China; jiamengyu@gs.zzu.edu.cn

**Keywords:** certificateless aggregate signature, conditional privacy-preserving, pseudonym, VANETs

## Abstract

Vehicular ad hoc networks (VANETs) have become a vital component of intelligent transport systems, with their security concerns increasingly drawing attention. To safeguard user privacy and ensure data authenticity and integrity, researchers have devised numerous certificateless conditional privacy-preserving authentication (CLCPPA) schemes. However, existing schemes generally suffer from insufficient security or high computational and communication overhead. Moreover, most implicitly assume the existence of a secure channel between vehicles and trusted entities during pseudonym generation and transmission, making it difficult to meet the real-time demands and practical deployment requirements of VANETs. To address these issues, this paper constructs a certificateless aggregated conditional privacy-preserving authentication (CL-ACPPA) scheme under elliptic curve cryptography that does not require bilinear operations. Formal security analysis demonstrates that, under the Random Oracle Model and the elliptic curve discrete logarithm problem assumption, the proposed scheme resists adaptive chosen-message attacks from adversaries with varying capabilities. Performance analysis and experimental results demonstrate that, compared with existing schemes, the proposed scheme achieves higher security while maintaining low communication and computational overhead.

## 1. Introduction

Within the context of intelligent transportation systems, VANETs have attracted considerable attention due to their great potential in enhancing road safety, traffic efficiency, and the driving experience. By enabling real-time information exchange between vehicles (V2V) and between vehicles and infrastructure (V2I), VANETs facilitate dynamic perception and sharing of traffic conditions. This contributes to accident prevention, congestion mitigation, and the optimization of overall traffic management [1,2,3]. However, owing to the highly mobile, open, and wireless nature of the vehicle networking environment, the communication process is susceptible to security attacks such as eavesdropping, tampering, and forgery. These threats compromise the confidentiality, authenticity, and integrity of information exchanged during vehicle communications, potentially jeopardizing traffic safety [4,5].

Thus, whilst safeguarding communication security within the vehicle-to-everything (V2X) network, it is imperative to implement vehicle privacy protection. Design solutions incorporating message authentication and anonymity can effectively fulfil these requirements. Presently, conditional privacy-preserving authentication (CPPA) schemes [6,7,8] have gained widespread adoption, ensuring both security and privacy through message authentication and vehicle anonymization techniques. Specifically, within CPPA frameworks, digital signature technology safeguards the authenticity, integrity, and validity of transmitted messages, while anonymization mechanisms enable conditional privacy protection, permitting the tracking of malicious vehicles’ true identities when necessary. To date, diverse CPPA schemes have been developed, including those based on public key cryptography (PKC-based) [9], identity-based cryptography (IBC-based) [10], and certificateless cryptography (CLC-based) [11,12,13]. CLC was proposed by Al-Riyami et al. [14] in 2003, wherein the Key Generation Center (KGC) distributes only partial private keys to users, who then combine these with their own selected secret values to form complete private keys. The KGC’s possession of only partial keys, without access to the complete key, effectively circumvents the key escrow issues inherent in IBC systems. As the CLC scheme eliminates certificate usage, it resolves challenges faced by PKC systems in certificate management and scalability. Consequently, it is particularly well-suited for resource-constrained vehicle networking environments. Designing CLCPPA schemes for vehicle networking has thus become a prominent research focus.

In VANETs, vehicle nodes require mutual authentication and generate a vast number of distinct signatures. During periods of heavy traffic, vehicles must validate numerous messages and signatures, imposing a significant computational burden. To address these challenges, Boneh et al. [15] pioneered the concept of aggregate signatures. By consolidating *n* signatures into a single compact signature, this approach enables batch verification, thereby reducing computational and communication costs. Consequently, integrating aggregate signatures into the CLCPPA scheme can enhance its efficiency. While numerous previous studies [16,17] have conducted research and design work on the CLCPPA scheme, its security remains insufficient against certain attack vectors. In 2021, Mei et al. [18] proposed the CLCPPA scheme for VANETs based on bilinear pairing. This scheme defends against attacks launched by malicious attackers capable of substituting vehicle public keys (Type-1) and malicious yet passive Key Generation Centers (Type-2) under the CLC security model. However, the scheme remained inefficient due to its reliance on costly bilinear pairings and Map-to-Point hash operations. Subsequently, in 2021, Ali et al. [19] and in 2025, Yang et al. [20] both proposed efficient, provably secure CLCPPA schemes for V2V communication based on elliptic curve cryptography. These schemes featured batch verification capabilities and eliminated the use of bilinear pairings. Nevertheless, Zhou et al. [21] subsequently demonstrated that Ali et al.’s scheme is vulnerable to signature forgery attacks, where both Type-1 and Type-2 attackers can successfully fabricate signatures, and developed a more secure CLCPPA scheme. In 2023, Gong et al. [22] designed an aggregated CLCPPA scheme to address vulnerabilities in Liu’s scheme. However, both Wu et al. [23] and Xu et al. [24] demonstrated that this scheme remains vulnerable to signature forgery attacks by Type-1 attackers. Notably, none of the aforementioned schemes considered pseudonym tampering attacks.

In 2023, Xiong et al. [25] proposed a conditionally privacy-preserving batch authentication scheme based on certificate-free aggregate signatures. This scheme leverages ECC structures to circumvent resource-intensive bilinear pairings. Analysis in this paper indicates that, while the scheme of Xiong et al. [25] resists injection attacks and signature-swapping attacks, the introduced parameter $\beta_{j}$ necessitates *n* scalar multiplication operations, resulting in a significant increase in computational complexity. This complexity even exceeds the time required to perform *n* separate signature verifications, essentially negating the efficiency advantage of batch verification. Furthermore, this paper observes that, during the local private key generation phase, the KGC merely treats the received pseudonym $PID_{i}$ as a hash input in the computation $Q_{i}=H_{1}\left(A_{i}\;\|\;PID_{i}\right)$, without performing any validity checks on $PID_{i}$ or consistency verification against the records of the Trusted Registration Authority (TRA). Consequently, should the pseudonym transmitted by the vehicle be tampered with during the vehicle–KGC communication process to become $PID_{i}^{\prime }$, the KGC will still generate partial private keys based on $PID_{i}^{\prime }$ and return them to the vehicle. The vehicle can only verify computational correctness via the equation $psk_{i}P=A_{i}+Q_{i}P_{\mathrm{KGC}}$, but cannot detect whether the pseudonym has been altered. This renders the scheme vulnerable to pseudonym tampering attacks.

In 2024, Kabil et al. [26] introduced an identity authentication module based on Chameleon hashes, aiming to enhance the robustness of vehicle authentication. They sought to improve resistance against pseudonym abuse/tampering through a two-stage verification process combining TRA and KGC. However, as the verification failed to cover the critical identity binding component $ID_{i,2}$, it remained ineffective against pseudonym tampering attacks. More recently, Xu et al. [27] proposed an improved CLCPPA scheme, which supports batch verification and is constructed based on ECC to achieve lightweight characteristics. Regrettably, analysis indicates this scheme fails to address injection attacks and signature-swapping attacks. Furthermore, during pseudonym transmission, the scheme assumes open wireless communication between vehicles and KGC/TRA without explicitly requiring secure channels. It also lacks integrity and consistency checks for received pseudonyms $PID_{i}$, enabling attackers to tamper with pseudonyms during transmission. As the KGC still participates in subsequent key generation or verification processes based on the tampered $PID_{i}$, the scheme struggles to resist pseudonym tampering attacks and fails to meet the requirement for conditional traceability. Although Ye et al. [28] introduced the Small Exponent Test (SET) to resist injection and signature-swapping attacks during batch verification, this scheme still assumes secure channels between vehicles and the TRA/KGC for transmitting pseudonyms and related information. Similarly, Wu et al. [29] rely on secure channels during pseudonym distribution. However, this paper contends that achieving permanent, perfect end-to-end secure channels is highly challenging.

In summary, the existing CLCPPA scheme suffers from the following issues:

- Existing solutions rely heavily on bilinear pairing operations with high computational overhead, resulting in elevated computational and communication costs in high-traffic scenarios. This makes it difficult to meet the requirements for low latency and high throughput.
- Existing approaches either implicitly assume secure communication channels between vehicles and trusted entities during pseudonym generation and transmission, or fail to implement adequate pseudonym validation over open channels. Consequently, they prove ineffective against pseudonym tampering attacks in weak or open-channel environments, falling short of the security and practical deployment requirements for VANETs.
- Certain existing approaches merely perform simple signature aggregation during batch verification. Attackers can exploit misalignment or cancellation to integrate invalid signatures into the batch, which may still pass verification due to the overall equation remaining valid. This renders the system vulnerable to injection attacks and signature-swapping attacks, compromising conditional traceability.

Consequently, designing a certificateless conditional privacy-preserving authentication scheme that simultaneously resists pseudonym tampering attacks and supports efficient aggregated verification holds significant research value, particularly without assuming a fully secure channel between vehicles and TRA/KGC. To address these challenges, this paper proposes a CL-ACPPA scheme based on ECC. The main contributions of the paper are summarized as follows.

- We achieve end-to-end verifiable binding of pseudonyms by having TRA sign vehicle pseudonyms. Before issuing a partial private key, the KGC mandatorily verifies the TRA signature using the corresponding public key. This effectively resists pseudonym tampering attacks under weak channel models, ensuring system security and conditional traceability in the event of accidents or disputes.
- We integrate the established Small Exponent Test (SET) into the proposed certificateless aggregate signature framework. By applying random small coefficients $s_{i}$ to individual signature components, the scheme prevents the algebraic cancellation exploited by injection and signature-swapping attacks. Unlike prior SET-based approaches, the contribution lies in combining this existing technique with TRA-authenticated pseudonyms, mandatory KGC-side verification, and RSU-assisted signature aggregation under open-channel conditions.
- We formally establish the correctness of individual and aggregate signature verification and analyze the unforgeability of the proposed scheme under the Random Oracle Model and the elliptic curve discrete logarithm problem assumption. We further examine resistance to pseudonym tampering, public key replacement, injection and signature-swapping attacks, and explicitly account for the computational cost of SET-based aggregation in the performance evaluation.

The remainder of this paper is organized as follows. Section 2 introduces the preliminaries.Section 3 provides the proposed scheme in detail. The security analysis of this scheme is given in Section 4. In Section 5, the scheme is analyzed in terms of computation and communication costs and it is compared with existing schemes. Section 6 discusses deployment considerations and the limitations of conditional traceability. Finally, the paper is concluded in Section 7.

## 2. Preliminaries

This section introduces some relevant foundational knowledge that will be utilized throughout this paper, including the elliptic curve discrete logarithm problem (ECDLP), system architecture, security models, and security requirements.

### 2.1. Elliptic Curve Discrete Logarithm Problem

Let *p* be a prime and $F_{p}$ the finite field. Then, an elliptic curve *E* over $F_{p}$ is defined by $E\left(a,b\right):y^{2}=\left(x^{3}+ax+b\right)\;\mathrm{mod}\;p$, where $a,b\in F_{p}$, and they satisfy $4a^{3}+27b^{2}\neq 0$. Within E(a,b), an additive cyclic group *G* of prime order *q* with generator *P* can be chosen.

The provable security of the proposed CL-ACPPA scheme reduces to ECDLP, whose precise definition is provided in Definition 1 below.

**Definition 1**  (ECDLP)**.** Given Q=xP in G, where $x\in Z_{q}^{*}$, the ECDLP is to find x from (P, Q). It is computationally hard if no probabilistic polynomial time (PPT) algorithm can solve it with non-negligible probability.

### 2.2. Small Exponent Test (SET)

The SET technique was originally proposed by Bellare et al. [30] to prevent malicious interference from multiple input instances during batch modular verification. SET is defined as follows:

**Definition 2**  (SET)**.** *Given n instances $\left(x_{i},Y_{i}\right)$, where $Y_{i}=x_{i}P$ and i=1,2,⋯,n. The verifier may select n distinct coefficients $s_{i}$(i=1,2,⋯,n), each of length l bits, to verify the validity of these n instances via equation $\left(\sum_{i=1}^{n}s_{i}x_{i}\right)P=\sum_{i=1}^{n}s_{i}Y_{i}$. The probability of an attacker correctly guessing any $s_{i}$ is less than $2^{-\ell }$, where $s_{i}\in \left[1,2^{\ell }\right]$.*

The purpose of SET is to prevent malicious interference from input instances by employing a small random generator between adjacent instances. According to Definition 2, if an injection and signature-swapping attacker launches an attack (e.g., corrupting instances $\left(x_{1}+\zeta ,Y_{1}\right)$ $\left(x_{2}-\zeta ,Y_{2}\right)$, where $\zeta \in Z_{q}^{*}$ and n=2), the batch verification equation $\left(s_{1}x_{1}+s_{1}\zeta +s_{2}x_{2}-s_{2}\zeta \right)P=s_{1}Y_{1}+s_{2}Y_{2}$ fails because $s_{1}\neq s_{2}$. The verifier will detect this attack via SET technology, and the attacker’s probability of compromising the batch verification equation’s equivalence validity is less than $2^{-\ell }$. This technique is primarily employed in Section 3 on aggregated signatures and batch verification.

### 2.3. Communication and Channel Model

During system initialization or offline vehicle registration, each vehicle’s OBU obtains the authentic system parameters, including the public keys of the TRA, KGC, and authorized RSUs. During online pseudonym generation, the vehicle selects an available RSU and constructs the forwarding path $V_{i}$-RSU-TRA. The vehicle encrypts its identity information using the published ECC public keys and transmits the resulting ciphertext through the open wireless channel. Therefore, the proposed scheme requires authentic system public parameters but does not require a pre-established confidential channel between the vehicle and the TRA/KGC.

Because the online wireless channel is open, an adversary may intercept, modify, delay, replace, or replay the transmitted messages. In the proposed scheme, the vehicle’s real identity is protected using ECC-based layered encryption. The authenticity and integrity of the pseudonym are protected by the TRA’s digital signature and the mandatory verification performed by the vehicle and KGC. The validity period $T_{i}$ and message timestamp $t_{i}$ are used to detect expired or replayed messages.

### 2.4. System Architecture

**Figure 1:**
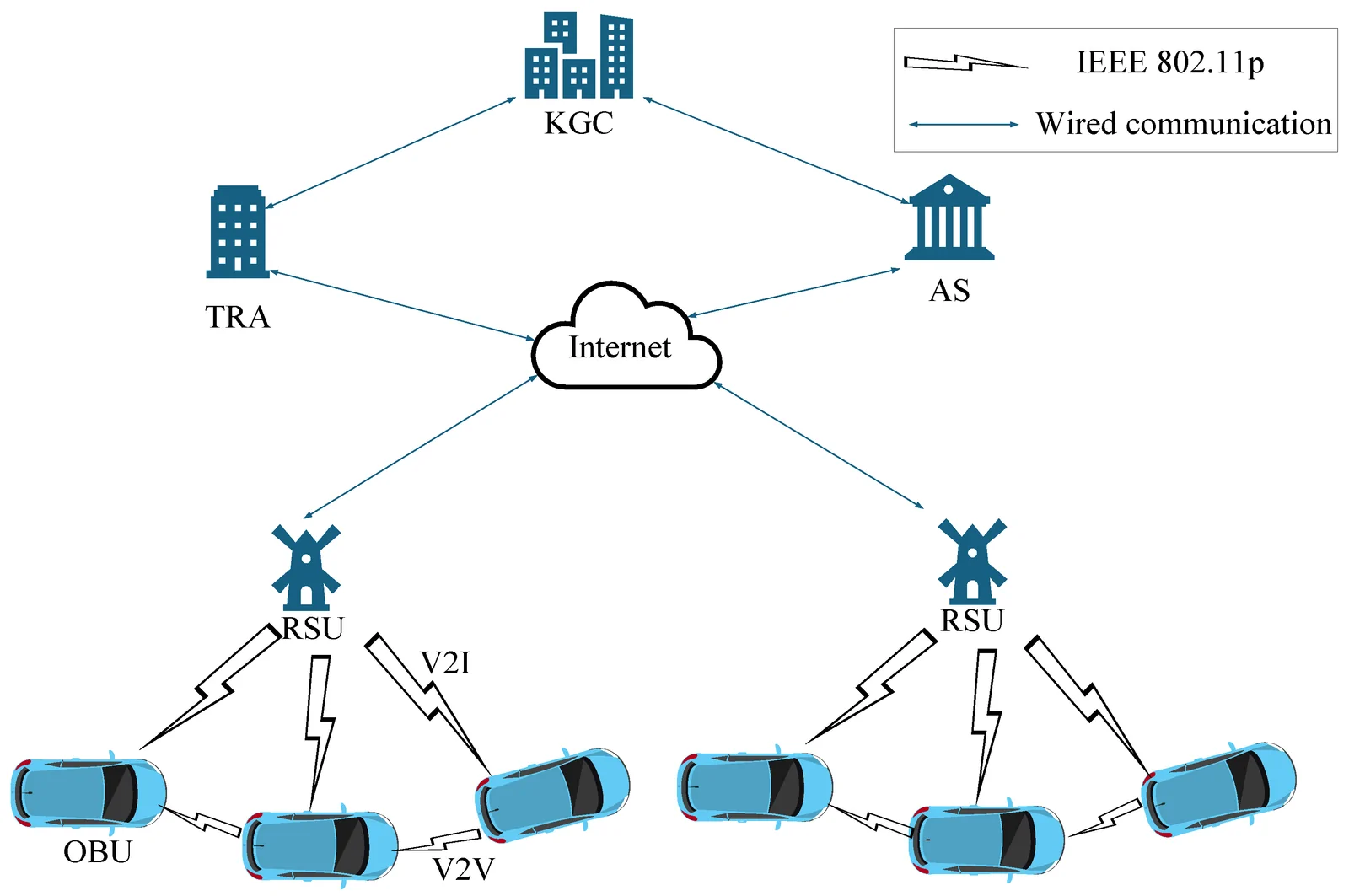
System architecture of VANETs.

As illustrated in Figure 1, VANETs typically comprise five entities: (1) Trusted Registration Authority (TRA); (2) Key Generation Center (KGC); (3) Roadside Unit (RSU); (4) Vehicle equipped with On-Board Unit (OBU); and (5) Application Server (AS). Detailed descriptions of each entity follow.

TRA: It is responsible for initializing the VANET system and completing vehicle registration, creating an anonymous identity $PID_{i}$ for each vehicle. Upon detecting malicious vehicles, the TRA tracks their true identities.

KGC: Upon receiving a vehicle’s $PID_{i}$, it generates the vehicle’s local private key and distributes it to the vehicle. Subsequently, at the vehicle end, the local private key combines with the vehicle key to generate signatures, ensuring VANET security.

RSU: Comprising base stations and Roadside Units, these possess greater processing capacity than On-Board Units and are responsible for consolidating signature data from vehicles. Additionally, RSU can generate the aggregate signatures to forward to the Application Server.

Vehicles: Each vehicle incorporates tamper-resistant OBU to prevent attackers from stealing stored data. Vehicles serve as the primary communication carriers, playing a crucial role. Each vehicle uses a unique key pair to sign messages, ensuring the confidentiality and legitimacy of transmitted data.

AS: This system is responsible for verifying the aggregate signature from RSU and assists in collecting and analyzing traffic data to predict traffic patterns, thereby enabling more efficient traffic signal control.

### 2.5. Security Model and Security Requirements

#### 2.5.1. Security Model

In 2005, Huang et al. [31] defined a corresponding security model based on practical application scenarios, identifying two classes of attackers (Type-1 and Type-2) and their respective capabilities. Certificateless signature schemes typically face attacks from two types of adversaries: malicious signers and malicious yet passive KGC. It is generally accepted that a certificateless signature scheme is deemed secure and satisfies non-repudiation if it successfully resists both attack types. The capabilities of these attackers are described as follows:

Type-1 attacker $A_{1}$ mimics a malicious vehicle within VANETs, capable of replacing vehicle public keys but unable to obtain the KGC’s master private key.

Type-2 attacker $A_{2}$ mimics a malicious yet passive KGC, capable of obtaining the KGC master private key but unable to replace any vehicle public keys.

Specifically, attackers $A_{1}$ and $A_{2}$ engage in simulated interactions with challengers $C_{1}$ and $C_{2}$ in Games 1 and 2, respectively. Within each game, adversary *A* may initiate the following oracle queries to *C*:

Public key query: the adversary queries an identity $PID_{i}$. *C* searches the maintained list for the identity. If present, it returns the user’s public key. If absent, it generates the user’s partial private key, secret value, and complete public key record in the list, then returns the user’s public key to the adversary.

Hash query: the adversary inputs any value for a hash query. *C* consults the list for the corresponding hash value. If present, the hash value is returned to the adversary. If absent, the hash value is computed, stored in the list, and returned to the adversary.

Partial private key query: on a partial private key query for $PID_{i}$, *C* returns it from the list if present; otherwise, *C* executes the user generation algorithm and returns the generated key.

Public key replacement query: the adversary performs a public key replacement query on $PID_{i}$. *C* replaces the public key corresponding to $PID_{i}$ based on the list.

Secret value query: on a secret value query for $PID_{i}$, *C* returns the value from the list if present; otherwise, *C* runs an initialization algorithm to generate and return it.

Signature query: the adversary issues a signature query for $PID_{i}$ and *M*. *C* signs the identity $PID_{i}$ and message *M* using the list and returns the signature to the adversary.

Batch signature query: *A* inputs *n* sets of signature messages $\left(sig_{i},t_{i},PID_{i},M_{i},pk_{i}\right)$ (i=1,2,…,n). *C* then verifies these *n* signatures and returns the results. Game 1: this game is conducted jointly by challenger $C_{1}$ and adversary $A_{1}$.

Initialization phase: $C_{1}$ executes the algorithm to generate system parameter params and the master key pair, sending params and the public key to $A_{1}$ while keeping the private key confidential.

Query phase: the adversary adaptively conducts user-generated interrogations, hash interrogations, partial private key interrogations, public key substitution interrogations, secret value interrogations, and signature interrogations based on the aforementioned capabilities.

Forging phase: ultimately, $A_{1}$ outputs a forged signature $sig^{*}$ using $PID_{i}^{*}$ and message *M*. $A_{1}$ wins the game if the following conditions are satisfied:

(a) $sig^{*}$ passes verification.
(b) $A_{1}$ has not performed any partial private key queries on $PID_{i}^{*}$.
(c) $A_{1}$ has not performed any signature queries on $PID_{i}^{*}$ or the signature $sig^{*}$ of *M*.

Game 2: this game is jointly conducted by $C_{2}$ and $A_{2}$.

Initialization phase: $C_{2}$ generates system parameter params and a master key pair and transmits them to $A_{2}$.

Query phase: the adversary adaptively performs user-generated queries, hash queries, partial private key queries, secret value queries, and signature queries based on the aforementioned capabilities.

Forging phase: ultimately, $A_{2}$ outputs a forged signature $sig^{*}$ with identity $PID_{i}^{*}$ and message *M*. $A_{2}$ wins the game if the following conditions are satisfied:

(a) $sig^{*}$ passes verification.
(b) $A_{2}$ has not previously made a secret value query for $PID_{i}^{*}$.
(c) $A_{2}$ has not made any signature queries regarding the signature $sig^{*}$ of $PID_{i}^{*}$ and *M*.

**Definition 3**  (existential unforgeability against adaptively chosen-message attacks, EUF-CMA)**.** *If no adversaries $A_{1}$ and $A_{2}$ exist who can win any of the two games above with a non-negligible advantage in polynomial time, then the proposed scheme satisfies EUF-CMA for Type-1 and Type-2 attacks.*

#### 2.5.2. Security Requirements

To ensure the security of VANET systems, the solution must satisfy the following security requirements.

- Message authentication. By validating signatures, the recipient ensures that messages originate from authorized vehicles and remain intact.
- Identity anonymity. Vehicles should employ pseudonyms during communication to conceal their true identities from other vehicles.
- Conditional traceability. In the event of an incident or dispute, TRA must possess the capability to retrieve and trace the true identities of vehicles.
- Replay resistance. Prevent attackers from capturing and reusing valid data transmissions with timestamps to impersonate users.
- Injection resistance. Attackers must be unable to generate valid signatures for modified or newly constructed messages by injecting additional information into existing signatures.
- Resistance to signature-swapping attacks. Attackers must be prevented from swapping or mismatching valid signatures across different messages in aggregate verification scenarios (e.g., applying $\sigma_{1}$ to $\left({\mathrm{PID}}_{2},M_{2}\right)$ and $\sigma_{2}$ to $\left({\mathrm{PID}}_{1},M_{1}\right)$) to exploit the linear aggregation property of batch verification and pass overall validation.
- Resistance to passive eavesdropping attack. The scheme should prevent an external passive eavesdropper from recovering the vehicle’s real identity or other sensitive plaintext information from intercepted pseudonym generation requests.
- Resistance to pseudonym tampering attacks. Even if attackers intercept legitimate pseudonyms or control partial network nodes, they cannot modify, replace, or forge vehicle pseudonyms without the vehicle’s knowledge or authorization, nor cause the system to accept such alterations as the vehicle’s legitimate identity.
- Resistance to public key replacement attacks. The scheme should prevent a Type-I adversary from forging a valid signature by replacing a vehicle-generated public key component, even if the adversary knows the secret value corresponding to the replaced public key. All public key components should be cryptographically bound to the vehicle’s pseudonym, message, and signing key, such that a replaced public key cannot be used to generate a valid signature without the corresponding partial private key.

In VANETs, security threats may originate from both external and internal adversaries. Under the considered adversarial model, an adversary is assumed to be capable of eavesdropping on and analyzing communications among vehicles and between vehicles and RSUs. The adversary may also intercept, modify, or inject messages in an attempt to infer sensitive information, such as vehicle identities and location information.

### 2.6. Analysis of the Kabil Scheme

This section analyzes and demonstrates that Kabil et al. [26] fail to adequately guarantee users’ conditional privacy and traceability. We directly extract the pseudonym generation algorithm and pseudonym verification algorithm from the scheme for analysis.

Vehicle generates PID: the On-Board Unit selects a random number $k_{i}\in Z_{q}^{*}$, computes $ID_{i,1}=k_{i}P$, $ID_{i,2}=RID_{i}⊕H_{1}\left(k_{i}T_{\mathrm{KGC}}\;\|\;VT_{i}\right)$, then constructs the pseudonym: $PID_{i}=\left(ID_{i,1},ID_{i,2},VT_{i}\right)$.

TRA authenticates PID: after validating $RID_{i}=ID_{i,2}⊕H_{1}\left(k_{i}T_{\mathrm{KGC}}\;\|\;VT_{i}\right)$ is legitimate, TRA computes $D_{i}=C-x\cdot ID_{i,1}$ and returns $D_{i}$ to the vehicle.

Vehicle requests partial private key from KGC: when $V_{i}$ requests a partial private key from KGC, it sends $\left(PID_{i},D_{i}\right)$. KGC verifies that $D_{i}+x\cdot ID_{i,1}=C$. If this equality holds, the KGC accepts $\left(PID_{i},D_{i}\right)$ and generates the partial private $h_{2,i}=H_{2}\left(A_{i}\;\|\;ID_{i,2}\right)$, $ppk_{i}=\left(\alpha_{i}+s\cdot h_{2,i}\right)\;\mathrm{mod}\;q$ for that vehicle.

It is evident that the Chameleon hash equation $D_{i}+x\cdot ID_{i,1}=C$ solely involves $ID_{i,1}$. The $ID_{i,2}$ bearing genuine identity information is not verified by either the TRA or KGC within this module. Consequently, provided a pair $\left(ID_{i,1},D_{i}\right)$ generated by trapdoor *x* is presented, KGC will accept it regardless of the corresponding value of $ID_{i,2}$.

For instance, consider a scenario involving a malicious intermediary *A* capable of intercepting the message $\left(PID_{i},D_{i}\right)$ transmitted from vehicle $V_{i}$ to the KGC. Upon acquiring this message, *A* preserves the legitimate $\left(ID_{i},D_{i}\right)$ unchanged, merely substituting the alias’s $ID_{i,2}$ with a forged value $ID_{i,2}^{\prime }$. *A* then constructs a new alias $PID_{i}^{\prime }=\left(ID_{i,1},ID_{i,2}^{\prime },VT_{i}\right)$ and forwards $\left(PID_{i}^{\prime },D_{i}\right)$ to the KGC. As the KGC’s verification equation only involves $ID_{i,1}$ and $D_{i}$, and is independent of $ID_{i,2}$, the KGC will accept this forged alias $PID_{i}^{\prime }$ without any knowledge of the substitution and will generate the corresponding partial private key for it. Throughout this process, the vehicle remains unaware of the anonymous identity generated and utilized on its behalf, and cannot verify its correctness or authenticity. It is evident that this scheme fails to adequately safeguard user anonymity, as it permits external entities to arbitrarily construct or tamper with the vehicle’s anonymous identity without its knowledge or consent.

Furthermore, when TRA attempts to trace disputed messages, it will encounter failure. The stored tracking information $\left(k_{i},RID_{i},VT_{i}\right)$ corresponds only to the original pseudonym $PID_{i}^{\prime }$, where $ID_{i,2}=RID_{i}⊕H_{1}\left(k_{i},T_{\mathrm{KGC}}\;\|\;VT_{i}\right)$, while the forged $ID_{i,2}^{\prime }$ does not correspond to any record. Consequently, the system cannot correctly recover $RID_{i}$ from $PID_{i}^{\prime }$. This malicious vehicle, while possessing legitimate certificates and signing capabilities, effectively becomes untraceable. Thus, the scheme intends for KGC to verify TRA’s authentication of pseudonyms for generating partial private keys, yet the core privacy and traceability information of the pseudonym is entirely contained within another component, $ID_{i,2}$ (generated by real identity encryption). The fatal flaw lies in the verification process that completely omits $ID_{i,2}$.

Consequently, the Kabil scheme fails to fully realize conditional privacy and traceability: an adversary can circumvent the TRA by arbitrarily modifying the pseudonym component $ID_{i,2}$ without detection by the KGC.

## 3. CL-ACPPA Scheme

### 3.1. Proposed Scheme

Based on the aforementioned analysis, this section introduces a security-enhanced certificateless aggregate signature-based conditional privacy-preserving authentication scheme for VANETs. The notations utilized in the scheme are summarized in Table 1.

**Figure 2:**
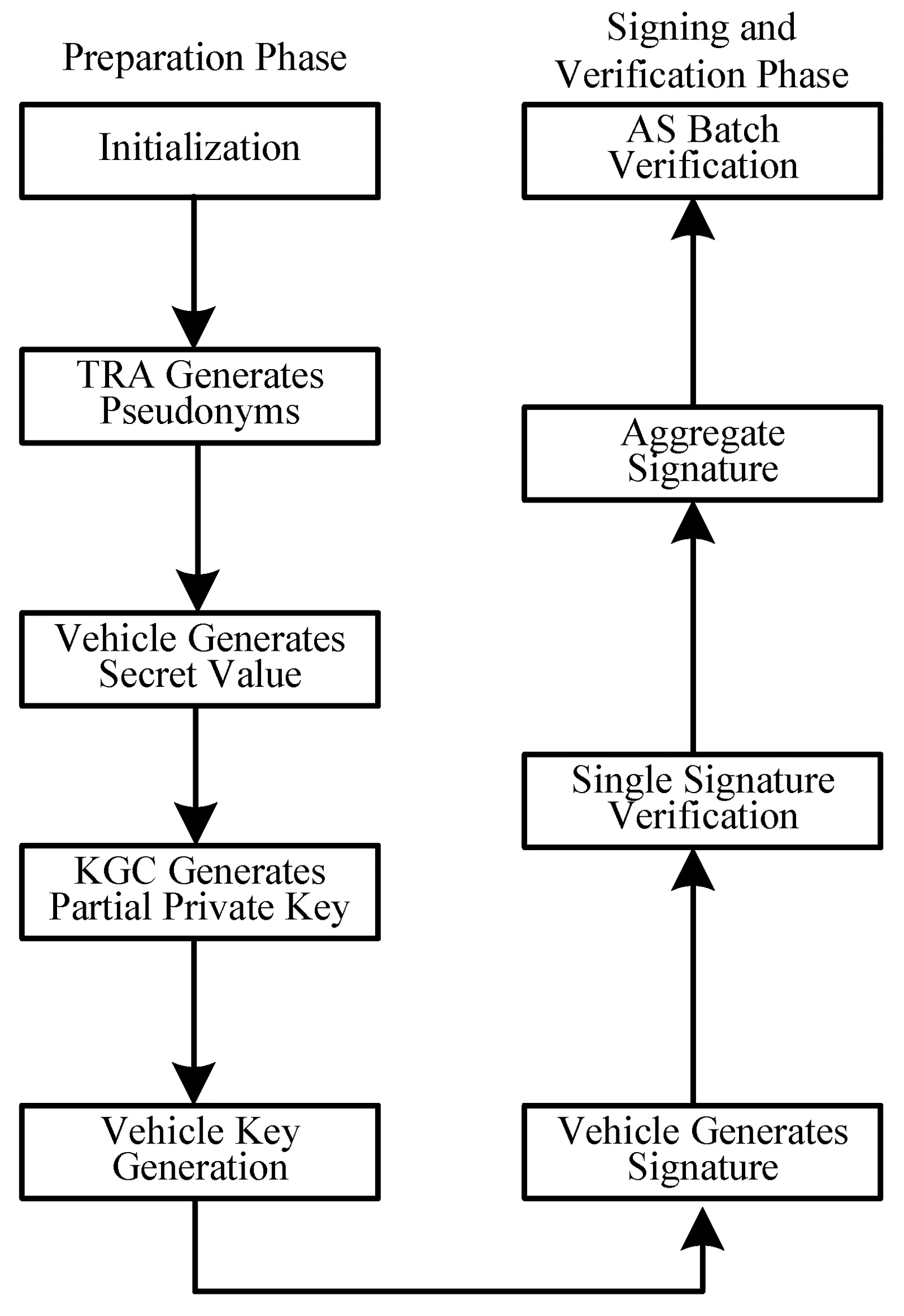
Workflow of the CL-ACPPA scheme.

The CL-ACPPA scheme can be divided into two phases based on algorithm functionality. The preparation phase includes the following algorithms: initialization, pseudonym generation, vehicle secret value generation, partial private key generation, and vehicle key generation. The signature and verification phase comprises individual signature generation, individual signature verification, aggregated signature, and batch verification. The algorithmic flow of the scheme is illustrated in Figure 2.

Initialization: TRA takes a security parameter *k* as input and generates a cyclic group *G* of prime order *q* with the generator *P*. It chooses a random number $\alpha \in Z_{q}^{*}$ as its secret key and computes $T_{TRA}=\alpha P$. Additionally, it selects three secure cryptographic hash functions $H_{0}:{\left\{0,1\right\}}^{*}\to {\left\{0,1\right\}}^{\lambda_{\mathrm{ID}}}$,$ID_{i}\in {\left\{0,1\right\}}^{\lambda_{\mathrm{ID}}}$, $H_{1}:{\left\{0,1\right\}}^{*}\times G\times G\times G\to Z_{q}^{*}$, $H_{2}:{\left\{0,1\right\}}^{*}\times {\left\{0,1\right\}}^{*}\times G\times G\times {\left\{0,1\right\}}^{*}\to Z_{q}^{*}$. KGC selects a random number $\beta \in Z_{q}^{*}$ as its secret key and computes $P_{\mathrm{KGC}}=\beta P$. TRA distributes key pairs $\left(r_{i},R_{i}\right)$ to each  RSU, where $R_{i}=r_{i}P$. Finally, the system parameters $G,q,P,H_{0},H_{1},H_{2},P_{\mathrm{KGC}},T_{TRA},R_{i}$ are published within the VANETs. Following initialization, each vehicle obtains a valid pseudonym and the corresponding TRA signature through the pseudonym generation procedure presented in Algorithm 1.
**Algorithm 1** Pseudonym generation**Input:** Real identity $ID_{i}$, system parameters params, TRA public key $T_{TRA}$, RSU public key $R_{i}$. **Output:** Vehicle pseudonym $PID_{i}$ and its signature $\sigma_{t}$. **Steps:** Before sending a message, a forwarding path $V_{i}\to RSU\to TRA$ is constructed, and the pseudonym generation request is forwarded along this path.
1.$V_{i}$ generates the inner authenticated ciphertext $c_{1}={\mathrm{Enc}}_{T_{\mathrm{TRA}}}\left(ID_{i}\right)=\left(K_{i},N_{1},C_{1},\tau_{1}\right),$ where $K_{i}=k_{i}P$ is the ephemeral public point of the inner encryption layer.2.$V_{i}$ serializes $c_{1}$ and generates the outer ciphertext $c_{2}={\mathrm{Enc}}_{R_{i}}\left(\mathrm{Serialize}(c_{1})\right).$ The randomness used in the inner and outer encryption layers is independently selected. $V_{i}$ sends $c_{2}$ to RSU.3.RSU computes ${\tilde{c}}_{1}={\mathrm{Dec}}_{r_{i}}\left(c_{2}\right).$ If ${\tilde{c}}_{1}=⊥$, the RSU rejects the request. Otherwise, it parses $c_{1}=\mathrm{Parse}\left({\tilde{c}}_{1}\right)$ and forwards $c_{1}$ to the TRA.4.The TRA computes $ID_{i}={\mathrm{Dec}}_{\alpha }\left(c_{1}\right).$ If the result is ⊥, the TRA rejects the request.5.TRA generates the vehicle pseudonym: computes $PID_{i,1}=ID_{i}⊕H_{0}(T_{i}\;\|\;EncodePoint\left(\alpha K_{i}\right)$), and the vehicle pseudonym is defined as $PID_{i}=\left(PID_{i,1},T_{i}\right)$.6.TRA signs the pseudonym $PID_{i}$ to obtain $\sigma_{t}={\mathrm{Sig}}_{\alpha }\left(PID_{i}\right)$, and sends $\left(PID_{i},\sigma_{t}\right)$ back to $V_{i}$.Here, ${\mathrm{Enc}}_{Q}\left(\cdot \right)$ and ${\mathrm{Dec}}_{s}\left(\cdot \right)$ denote ECC-based authenticated hybrid encryption and decryption, respectively, where Q=sP. The shared point derived from ephemeral ECDH is converted by a KDF into a symmetric key for authenticated encryption; decryption returns the plaintext if authentication succeeds and ⊥ otherwise.

Vehicle secret value generation: after receiving $PID_{i}$, the vehicle first verifies whether $ID_{i}=PID_{i,1}⊕H_{0}\left(T_{i}\;\|\;\mathrm{EncodePoint}\left(k_{i}T_{\mathrm{TRA}}\right)\right)$ is correct (ensuring that the received pseudonym is generated by TRA and not forged). Then, it randomly selects a secret value $x_{i}\in Z_{q}^{*}$ and computes $X_{i}=x_{i}P$.

Partial private key generation: the vehicle $V_{i}$ sends the partial private key request $\left(PID_{i},\sigma_{t},X_{i}\right)$ to the KGC. Upon receiving the request, the KGC executes the following steps in the specified order.

1. Mandatory pseudonym verification: the KGC first checks the validity period $T_{i}$ and verifies the TRA signature using the authenticated TRA public key $T_{\mathrm{TRA}}$: ${\mathrm{Ver}}_{T_{\mathrm{TRA}}}\left(PID_{i},\sigma_{t}\right)\overset{?}{=}1\;$. This verification is a mandatory prerequisite for partial private key generation. If the signature verification fails or $T_{i}$ has expired, the KGC immediately outputs ⊥, rejects the request, and terminates the algorithm without generating $y_{i}$, $Y_{i}$, $h_{1,i}$, or $d_{i}$.
2. Partial private key computation: only if the preceding verification succeeds does the KGC randomly select $y_{i}\in Z_{q}^{*}$, compute $Y_{i}=y_{i}P,$ $h_{1,i}=H_{1}(PID_{i}∥Y_{i}∥P_{\mathrm{KGC}}∥X_{i}),$ and generate $d_{i}=y_{i}+h_{1,i}\beta$. The KGC then sends $\left(d_{i},Y_{i}\right)$ to $V_{i}$.
3. Vehicle-side correctness verification: after receiving $\left(d_{i},Y_{i}\right)$, $V_{i}$ recomputes $h_{1,i}$ and verifies $\;d_{i}P\overset{?}{=}Y_{i}+h_{1,i}P_{\mathrm{KGC}}\;$. If the equation does not hold, $V_{i}$ rejects the partial private key and submits a new request. This process binds $V_{i}$’s pseudonym $PID_{i}$ to the partial public key $X_{i}$ generated from the selected secret value.

Because $\sigma_{t}$ covers the complete pseudonym $PID_{i}$, any modification of $PID_{i,1}$ or $T_{i}$ invalidates the TRA signature. Therefore, an adversary that replaces $PID_{i}$ with $PID_{i}^{\prime }$ must also generate a valid signature $\sigma_{t}^{\prime }$ satisfying

(1) $$\begin{array}{c} {\mathrm{Ver}}_{T_{\mathrm{TRA}}}\left(PID_{i}^{\prime },\sigma_{t}^{\prime }\right)=1\; \end{array}$$

which is equivalent to forging the TRA’s digital signature. Consequently, the KGC generates a partial private key only for a valid and unmodified pseudonym issued by the TRA.

Vehicle key generation: the private key of $V_{i}$ is $sk_{i}=\left(d_{i},x_{i}\right)$, and the public key is $pk_{i}=\left(X_{i},Y_{i},pk_{2,i}\right)$, where $pk_{2,i}=Y_{i}+h_{1,i}X_{i}$.

Signature generation: $V_{i}$ generates a signature for message $M_{i}$ through the following algorithm:

(1) $V_{i}$ randomly selects $z_{i}\in Z_{q}^{*}$ and computes $Z_{i}=z_{i}P$, $h_{2,i}=H_{2}(M_{i}\;\|\;PID_{i}\;\|\;pk_{i}\;\|\;Z_{i}\;\|\;t_{i})$, where $t_{i}$ is a timestamp. $V_{i}$ computes $\sigma_{i}=z_{i}+h_{2,i}\left(h_{1,i}x_{i}+d_{i}\right)$, and outputs the signature $sig_{i}=\left(Z_{i},\sigma_{i}\right)$.
(2) $V_{i}$ sends the message $\left(sig_{i},t_{i},PID_{i},M_{i},pk_{i}\right)$ to the verifying RSU or other vehicle units.

Signature verification: after receiving the message (sig_*i*_, $t_{i}$, $PID_{i}$, $M_{i}$, $pk_{i}$) from $V_{i}$, the RSU performs verification through the following algorithm: verify whether $t_{i}$ is valid. If so, compute $h_{1,i}=H_{1}(PID_{i}\;\|\;Y_{i}\;\|\;P_{\mathrm{KGC}}\;\|\;X_{i})$ and $h_{2,i}=H_{2}(M_{i}\;\|\;PID_{i}\;\|\;pk_{i}\;\|\;Z_{i}\;\|\;t_{i})$, and then verify whether $\sigma_{i}P=Z_{i}+h_{2,i}\left(pk_{2,i}+h_{1,i}P_{\mathrm{KGC}}\right)$ holds. If it holds, accept the signature; otherwise, reject it.

Aggregate signature: after receiving *n* authentication messages (sig_*i*_, $t_{i}$, $PID_{i}$, $M_{i}$, $pk_{i}$), RSU first verifies the validity of the timestamps. It then randomly selects distinct SET coefficients $s_{i}\in \left[1,2^{\ell }\right],i=1,2,\ldots ,n,$ and computes $\sigma =\sum_{i=1}^{n}s_{i}\sigma_{i}$ and $Z=\sum_{i=1}^{n}s_{i}Z_{i}.$

For each message, RSU constructs the verification context $C_{i}=\left(s_{i},Z_{i},t_{i},PID_{i},M_{i},pk_{i}\right).$ The complete aggregate verification packet is defined as $\Pi =\left(\sigma ,Z,{\left\{C_{i}\right\}}_{i=1}^{n}\right).$ The RSU sends Π to the AS.

Batch verification: when AS receives Π: first, verify whether each $t_{i}$ is valid. If all are valid, then verify whether

(2) $$\begin{array}{c} \sigma P\overset{?}{=}Z+\sum_{i=1}^{n}s_{i}h_{2,i}\left(pk_{2,i}+h_{1,i}P_{\mathrm{KGC}}\right) \end{array}$$

If the equation holds, the AS accepts the batch; otherwise, it rejects the batch.

### 3.2. Correctness Analysis

#### 3.2.1. Individual Signature Verification

When a verifier receives a message (sig_*i*_(Z_*i*_, $\sigma_{i}$), $t_{i}$, $PID_{i}$, $M_{i}$, $pk_{i}$) from $V_{i}$, the verifier checks whether the equation

(3) $$\begin{array}{c} \sigma_{i}\cdot P=Z_{i}+h_{2,i}\left(pk_{2,i}+h_{1,i}P_{\mathrm{KGC}}\right) \end{array}$$

holds. If it holds, the signature is considered valid.

**Proof.**  Given $\sigma_{i}=z_{i}+h_{2,i}\left(h_{1,i}x_{i}+d_{i}\right)$, the following derivation can be obtained:

(4) $$\begin{array}{cc} {}[z_{i}+h_{2,i}(h_{1,i}x_{i}+d_{i})]P & =z_{i}P+h_{2,i}\left(h_{1,i}x_{i}P+d_{i}P\right)\\ \; & =Z_{i}+h_{2,i}\left(h_{1,i}X_{i}+Y_{i}+h_{1,i}P_{\mathrm{KGC}}\right)\\ \; & =Z_{i}+h_{2,i}\left(pk_{2,i}+h_{1,i}P_{\mathrm{KGC}}\right)\\ \; & =\sigma_{i}P \end{array}$$

Therefore, the verification equation holds, and the signature is correct. □

#### 3.2.2. Batch Signature Verification

For *n* valid signatures ${\left\{\sigma_{i}\right\}}_{i=1}^{n}$, if the equation $\sigma P=Z+\sum_{i=1}^{n}s_{i}h_{2,i}\left(pk_{2,i}+h_{1,i}P_{\mathrm{KGC}}\right)$ holds, the batch of signatures is considered valid.

**Proof.**  Since $\sigma_{i}=z_{i}+h_{2,i}\left(h_{1,i}x_{i}+d_{i}\right)$, $pk_{2,i}=Y_{i}+h_{1,i}X_{i}$, and $d_{i}P=\left(y_{i}+h_{1,i}\beta \right)P=Y_{i}+h_{1,i}P_{\mathrm{KGC}}$, the following derivation can be obtained:

(5) $$\begin{array}{cc} \sigma \cdot P & =\sum_{i=1}^{n}s_{i}\sigma_{i}\cdot P\\ \; & =\sum_{i=1}^{n}s_{i}\left(z_{i}+h_{2,i}\left(h_{1,i}x_{i}+d_{i}\right)\right)P\\ \; & =Z+\sum_{i=1}^{n}s_{i}h_{2,i}\left(pk_{2,i}+h_{1,i}P_{\mathrm{KGC}}\right) \end{array}$$

Therefore, the verification equation holds and the signature is correct. □

## 4. Security Analysis

### 4.1. Security Proof

According to the definition of the security model in Section 2, this section demonstrates the security of our scheme through the game between a challenger 𝒞 and two types of adversaries.

**Theorem 1.**  *Under the Random Oracle Model(ROM), if the ECDLP assumption holds, the proposed scheme is EUF-CMA for a Type-I adversary $A_{1}$.*

**Proof.**  Assume $A_{1}$ is a PPT Type-I adversary capable of forging a valid signature of our scheme. We construct a challenger ${𝒞}_{1}$ that uses $A_{1}$ to solve an ECDLP instance (P,Q=λP), where $\lambda \in Z_{q}^{*}$ is unknown to ${𝒞}_{1}$. The interaction between ${𝒞}_{1}$ and $A_{1}$ is described as follows.

Setup: the challenger randomly selects the KGC master secret $\beta \in Z_{q}^{*}$ and computes $P_{\mathrm{KGC}}=\beta P.$ It then publishes the system parameters $\{G,q,P,H_{0},H_{1},H_{2},P_{\mathrm{KGC}},T_{\mathrm{TRA}},R_{i}\}.$ Therefore, ${𝒞}_{1}$ knows β, while the unknown ECDLP value λ remains hidden in *Q*.

The challenger guesses a target identity $PID_{i}^{*}$ and maintains the lists *L*, $L_{H_{1}}$, $L_{H_{2}}$, and $L_{S}$ to ensure the consistency of the public key, random oracle, and signing-query simulations.

For the target identity $PID_{i}^{*}$, ${𝒞}_{1}$ embeds the ECDLP instance by setting $Y_{i}^{*}=Q=\lambda P.$ It randomly selects $x_{i}^{*},h_{1,i}^{*}\in Z_{q}^{*}$ computes $X_{i}^{*}=x_{i}^{*}P$ and programs $H_{1}\left(PID_{i}^{*}∥Y_{i}^{*}∥P_{\mathrm{KGC}}∥X_{i}^{*}\right)=h_{1,i}^{*}.$ The public key of the target vehicle is defined as $pk_{i}^{*}=\left(X_{i}^{*},Y_{i}^{*},pk_{2,i}^{*}\right)=\left(X_{i}^{*},Y_{i}^{*},Y_{i}^{*}+h_{1,i}^{*}X_{i}^{*}\right).$ The corresponding partial private key would be $d_{i}^{*}=\lambda +h_{1,i}^{*}\beta \;\left(\mathrm{mod}\;q\right),$ which is unknown to ${𝒞}_{1}$. Nevertheless, as shown below, the challenger can simulate signing queries without knowing $d_{i}^{*}$.

Query phase:

(a) User generation queries: when $A_{1}$ submits a user generation query for $PID_{i}$, ${𝒞}_{1}$ first searches *L*. If a corresponding entry exists, it returns the stored public key.
  If $PID_{i}=PID_{i}^{*}$, ${𝒞}_{1}$ returns $pk_{i}^{*}$ generated above and stores $\left(PID_{i}^{*},X_{i}^{*},Y_{i}^{*},pk_{i}^{*},x_{i}^{*},⊥,h_{1,i}^{*}\right)$ in *L*, where ⊥ indicates that the target partial private key is unknown.
  If $PID_{i}\neq PID_{i}^{*}$, ${𝒞}_{1}$ generates the user normally. It randomly selects $x_{i},y_{i}\in Z_{q}^{*}$ computes $X_{i}=x_{i}P,Y_{i}=y_{i}P$ obtains $h_{1,i}=H_{1}\left(PID_{i}∥Y_{i}∥P_{\mathrm{KGC}}∥X_{i}\right)$ and calculates $d_{i}=y_{i}+h_{1,i}\beta .$ It then constructs $pk_{i}=\left(X_{i},Y_{i},Y_{i}+h_{1,i}X_{i}\right)$ and stores all the corresponding values in *L*.
(b) $H_{1}$ queries: when $A_{1}$ submits $\left(PID_{i},Y_{i},P_{\mathrm{KGC}},X_{i}\right)$, ${𝒞}_{1}$ searches $L_{H_{1}}$. If a corresponding entry exists, it returns the stored value. Otherwise, it randomly selects $h_{1,i}\in Z_{q}^{*},$ stores $\left(PID_{i},Y_{i},P_{\mathrm{KGC}},X_{i},h_{1,i}\right)$ in $L_{H_{1}}$, and returns $h_{1,i}$.
(c) $H_{2}$ queries: when $A_{1}$ submits $\left(M_{i},PID_{i},pk_{i},Z_{i},t_{i}\right)$ to $H_{2}$, ${𝒞}_{1}$ searches $L_{H_{2}}$. If the corresponding entry exists, it returns the stored value. Otherwise, it randomly selects $h_{2,i}\in Z_{q}^{*},$ stores $\left(M_{i},PID_{i},pk_{i},Z_{i},t_{i},h_{2,i}\right)$ in $L_{H_{2}}$ and returns $h_{2,i}$.
(d) Partial private key queries: when $A_{1}$ requests the partial private key corresponding to $PID_{i}$, ${𝒞}_{1}$ proceeds as follows. If $PID_{i}=PID_{i}^{*},$ the challenger aborts because it does not know $d_{i}^{*}=\lambda +h_{1,i}^{*}\beta \;\left(\mathrm{mod}\;q\right).$ If $PID_{i}\neq PID_{i}^{*}$, the challenger returns the corresponding $d_{i}$ stored in *L*. If no corresponding entry exists, it first executes a user generation query and then returns $d_{i}$.
(e) Public key replacement queries: when $A_{1}$ replaces $X_{i}$ with an arbitrary point $X_{i}^{\prime }$, ${𝒞}_{1}$ obtains or programs $h_{1,i}^{\prime }=H_{1}\left(PID_{i}∥Y_{i}∥P_{\mathrm{KGC}}∥X_{i}^{\prime }\right)$ and updates the public key as $pk_{i}^{\prime }=\left(X_{i}^{\prime },Y_{i},Y_{i}+h_{1,i}^{\prime }X_{i}^{\prime }\right).$ The challenger records that the secret value corresponding to $X_{i}^{\prime }$ is unknown. As usual in the Type-I security game, incompatible secret value extraction queries for an adversary-replaced target key are excluded from a valid forgery.
(f) Secret value queries: if the queried public key has not been replaced, ${𝒞}_{1}$ returns the corresponding $x_{i}$ stored in *L*. For a non-target identity that has not yet been generated, ${𝒞}_{1}$ first executes a user generation query and then returns $x_{i}$.
(g) Signature queries: for a non-target identity, ${𝒞}_{1}$ generates a signature according to the original signing algorithm.

For the target identity $PID_{i}^{*}$, the challenger does not know $d_{i}^{*}$. It therefore randomly selects $\sigma_{i},h_{2,i}\in Z_{q}^{*}$ and computes $Z_{i}=\sigma_{i}P-h_{2,i}\left(pk_{2,i}^{*}+h_{1,i}^{*}P_{\mathrm{KGC}}\right).$ It then programs the random oracle as $H_{2}\left(M_{i}∥PID_{i}^{*}∥pk_{i}^{*}∥Z_{i}∥t_{i}\right)=h_{2,i}.$ If the corresponding $H_{2}$ entry has already been defined inconsistently, the challenger repeats the simulation with fresh random values.

The simulated signature $sig_{i}=\left(Z_{i},\sigma_{i}\right)$ satisfies $\sigma_{i}P=Z_{i}+h_{2,i}\left(pk_{2,i}^{*}+h_{1,i}^{*}P_{\mathrm{KGC}}\right)$ and is therefore indistinguishable from a signature generated by the original signing algorithm.

Forgery phase: eventually, $A_{1}$ outputs a purported forgery $sig_{i}^{*}=\left(Z_{i}^{*},\sigma_{i}^{*}\right)$ on $\left(PID_{i},M_{i},pk_{i},t_{i}\right).$ The challenger aborts unless $PID_{i}=PID_{i}^{*}$. A successful forgery must satisfy the original verification equation $\sigma_{i}^{*}P=Z_{i}^{*}+h_{2,i}^{*}\left(pk_{2,i}^{*}+h_{1,i}^{*}P_{\mathrm{KGC}}\right)$ and $A_{1}$ must not have requested the target partial private key or a signature on the same message tuple.

The extraction is divided into the following two cases.

Case 1: the target public key has not been replaced.

By applying the Forking Lemma to the $H_{2}$ query, ${𝒞}_{1}$ obtains two valid forgeries $\left(Z_{i}^{*},\sigma_{i}^{*}\right)\;\mathrm{and}\;\left(Z_{i}^{*},{\hat{\sigma }}_{i}^{*}\right)$ with the same commitment $Z_{i}^{*}$, but with two distinct hash values $h_{2,i}^{*}\neq {\hat{h}}_{2,i}^{*}.$ Thus, $\sigma_{i}^{*}P=Z_{i}^{*}+h_{2,i}^{*}\left(pk_{2,i}^{*}+h_{1,i}^{*}P_{\mathrm{KGC}}\right)$ and ${\hat{\sigma }}_{i}^{*}P=Z_{i}^{*}+{\hat{h}}_{2,i}^{*}\left(pk_{2,i}^{*}+h_{1,i}^{*}P_{\mathrm{KGC}}\right).$ Subtracting these equations gives $\left(\sigma_{i}^{*}-{\hat{\sigma }}_{i}^{*}\right)P=\left(h_{2,i}^{*}-{\hat{h}}_{2,i}^{*}\right)\left(pk_{2,i}^{*}+h_{1,i}^{*}P_{\mathrm{KGC}}\right).$ Because $pk_{2,i}^{*}=Q+h_{1,i}^{*}X_{i}^{*}$, we obtain $pk_{2,i}^{*}+h_{1,i}^{*}P_{\mathrm{KGC}}=\left[\lambda +h_{1,i}^{*}\left(x_{i}^{*}+\beta \right)\right]P.$ Define $w_{i}=\left(\sigma_{i}^{*}-{\hat{\sigma }}_{i}^{*}\right){\left(h_{2,i}^{*}-{\hat{h}}_{2,i}^{*}\right)}^{-1}\;\left(\mathrm{mod}\;q\right).$ It follows that $w_{i}=\lambda +h_{1,i}^{*}\left(x_{i}^{*}+\beta \right)\;\left(\mathrm{mod}\;q\right).$ Therefore, the challenger extracts $\lambda =w_{i}-h_{1,i}^{*}\left(x_{i}^{*}+\beta \right).$

Case 2: the target public key has been replaced.

Suppose that $A_{1}$ replaces $X_{i}^{*}$ with $X_{i}^{\prime }=x_{i}^{\prime }P,$ where $x_{i}^{\prime }$ may be unknown to ${𝒞}_{1}$. The corresponding public key component is $pk_{2,i}^{\prime }=Q+h_{1,i}X_{i}^{\prime }.$ A single $H_{2}$-fork would only reveal $\lambda +h_{1,i}\left(x_{i}^{\prime }+\beta \right),$ which is insufficient because $x_{i}^{\prime }$ is unknown. Therefore, the challenger applies the generalized nested forking argument to the relevant $H_{1}$ and $H_{2}$ queries.

For a fixed $H_{1}$ response $h_{1,i}$, two $H_{2}$-forked forgeries give

(6) $$\begin{array}{c} w_{i}=\left(\sigma_{i,1}-\sigma_{i,2}\right){\left(h_{2,i,1}-h_{2,i,2}\right)}^{-1}=\lambda +h_{1,i}\left(x_{i}^{\prime }+\beta \right) \end{array}$$

The challenger then replays $A_{1}$ with the same random tape and the same replacement point $X_{i}^{\prime }$, but programs the relevant $H_{1}$ query with a different value ${\hat{h}}_{1,i}\neq h_{1,i}.$ Applying the $H_{2}$-fork again gives ${\hat{w}}_{i}=\left({\hat{\sigma }}_{i,1}-{\hat{\sigma }}_{i,2}\right){\left({\hat{h}}_{2,i,1}-{\hat{h}}_{2,i,2}\right)}^{-1}=\lambda +{\hat{h}}_{1,i}\left(x_{i}^{\prime }+\beta \right)$. Eliminating the unknown value $x_{i}^{\prime }+\beta$ from these two equations yields

(7) $$\begin{array}{c} \lambda =\left(h_{1,i}{\hat{w}}_{i}-{\hat{h}}_{1,i}w_{i}\right){\left(h_{1,i}-{\hat{h}}_{1,i}\right)}^{-1} \end{array}$$

Hence, even in the public key replacement case, ${𝒞}_{1}$ extracts the ECDLP solution without knowing either $y_{i}^{*}$ or $x_{i}^{\prime }$.

If $A_{1}$ succeeds with non-negligible probability, then, after the polynomial losses caused by guessing the target identity and applying the forking procedure, ${𝒞}_{1}$ also solves the ECDLP with non-negligible probability. This contradicts the assumed hardness of the ECDLP. Therefore, our scheme is existentially unforgeable against adaptive chosen-message attacks launched by a Type-I adversary. □

**Theorem 2.**  *Under the ROM, if ECDLP assumption holds, the proposed scheme is EUF-CMA for a Type-II adversary $A_{2}$.*

**Proof.**  Assume $A_{2}$ is a PPT Type-II adversary capable of forging a valid signature of our scheme. The challenger ${𝒞}_{2}$ takes an ECDLP instance Q=λP and intends to utilize $A_{2}$ to solve the ECDLP. ${𝒞}_{2}$ selects $PID_{i}^{*}$ as the anonymous challenge identity and $sig_{i}^{*}$ as the corresponding forged signature. The interaction between $A_{2}$ and ${𝒞}_{2}$ proceeds as follows:

*Setup:* the challenger randomly selects $\beta \in Z_{q}^{*}$ and computes $P_{\mathrm{KGC}}=\beta P.$ It generates and publishes $\mathrm{params}=\{G,q,P,H_{0},H_{1},H_{2},P_{\mathrm{KGC}},T_{\mathrm{TRA}},R_{i}\}$ and provides the master secret β to $A_{2}$.

The challenger guesses a target pseudonym $PID_{i}^{*}$ and initializes the lists *L*, $L_{H_{1}}$, $L_{H_{2}}$, and $L_{S}$ to maintain consistent responses to the adversary’s queries.

For the target pseudonym, the challenger embeds the ECDLP instance in the vehicle-generated public key component by setting $X_{i}^{*}=Q=\lambda P.$ It randomly selects $y_{i}^{*},h_{1,i}^{*}\in Z_{q}^{*}$ and computes $Y_{i}^{*}=y_{i}^{*}P.$ The challenger programs $H_{1}\left(PID_{i}^{*}∥Y_{i}^{*}∥P_{\mathrm{KGC}}∥X_{i}^{*}\right)=h_{1,i}^{*}$ and calculates $d_{i}^{*}=y_{i}^{*}+h_{1,i}^{*}\beta$ and $pk_{2,i}^{*}=Y_{i}^{*}+h_{1,i}^{*}X_{i}^{*}.$ Accordingly, the complete target public key is $pk_{i}^{*}=\left(X_{i}^{*},Y_{i}^{*},pk_{2,i}^{*}\right)=\left(Q,Y_{i}^{*},Y_{i}^{*}+h_{1,i}^{*}Q\right).$

Query phase:

(a) User generation queries: when $A_{2}$ submits a user generation query for $PID_{i}$, the challenger first searches *L*. If a matching entry exists, it returns the stored complete public key.
  If $PID_{i}=PID_{i}^{*}$, the challenger returns $pk_{i}^{*}$ and stores $\left(PID_{i}^{*},X_{i}^{*},Y_{i}^{*},pk_{2,i}^{*},⊥,d_{i}^{*},y_{i}^{*},h_{1,i}^{*}\right)$ in *L*, where ⊥ indicates that the target secret value $x_{i}^{*}=\lambda$ is unknown.
  If $PID_{i}\neq PID_{i}^{*}$, the challenger executes the normal user generation algorithm. It randomly selects $x_{i},y_{i}\in Z_{q}^{*}$ and computes $X_{i}=x_{i}P,Y_{i}=y_{i}P.$ It obtains $h_{1,i}=H_{1}(PID_{i}∥Y_{i}∥P_{\mathrm{KGC}}∥X_{i})$ and calculates $d_{i}=y_{i}+h_{1,i}\beta$ and $pk_{2,i}=Y_{i}+h_{1,i}X_{i}.$ The complete public key is $pk_{i}=\left(X_{i},Y_{i},pk_{2,i}\right).$ The challenger stores $\left(PID_{i},X_{i},Y_{i},pk_{2,i},x_{i},d_{i},y_{i},h_{1,i}\right)$ in *L* and returns $pk_{i}$.
(b) $H_{1}$ queries: when $A_{2}$ submits $\left(PID_{i},Y_{i},P_{\mathrm{KGC}},X_{i}\right)$ to $H_{1}$, the challenger searches $L_{H_{1}}$. If a matching entry exists, it returns the stored value. Otherwise, it randomly selects $h_{1,i}\in Z_{q}^{*},$ stores $\left(PID_{i},Y_{i},P_{\mathrm{KGC}},X_{i},h_{1,i}\right)$ in $L_{H_{1}}$, and returns $h_{1,i}$.
(c) $H_{2}$ queries: when $A_{2}$ submits $\left(M_{i},PID_{i},pk_{i},Z_{i},t_{i}\right)$ to $H_{2}$, the challenger searches $L_{H_{2}}$. If a matching entry exists, it returns the stored value. Otherwise, it randomly selects $h_{2,i}\in Z_{q}^{*},$ stores $\left(M_{i},PID_{i},pk_{i},Z_{i},t_{i},h_{2,i}\right)$ in $L_{H_{2}}$, and returns $h_{2,i}$.
(d) Partial private key queries: when $A_{2}$ requests the partial private key corresponding to $PID_{i}$, the challenger searches *L*. If an entry exists, it returns $d_{i}$. Otherwise, it first executes a user generation query and then returns $d_{i}$.
  In particular, the challenger can answer the target partial private key query by returning $d_{i}^{*}=y_{i}^{*}+h_{1,i}^{*}\beta$.
(e) Secret value queries: when $A_{2}$ requests the secret value corresponding to $PID_{i}$, the challenger checks whether $PID_{i}=PID_{i}^{*}.$ If this condition holds, the challenger aborts. Otherwise, it returns the corresponding $x_{i}$ stored in *L*. If no corresponding entry exists, it first executes a user generation query and then returns $x_{i}$.
(f) Signature queries: when $A_{2}$ requests a signature on $(M_{i},PID_{i},pk_{i},t_{i}),$ the challenger first obtains the corresponding complete public key $pk_{i}.$

For a non-target identity, the challenger executes the original signing algorithm because it knows the complete private key.

It randomly selects $\sigma_{i},h_{2,i}\in Z_{q}^{*}$ and computes $Z_{i}=\sigma_{i}P-h_{2,i}\left(pk_{2,i}^{*}+h_{1,i}^{*}P_{\mathrm{KGC}}\right).$ The challenger programs $H_{2}\left(M_{i}∥PID_{i}^{*}∥pk_{i}^{*}∥Z_{i}∥t_{i}\right)=h_{2,i}.$ If this $H_{2}$ input has already been assigned an inconsistent value, the challenger repeats the simulation using fresh random values.

The simulated signature $sig_{i}=\left(Z_{i},\sigma_{i}\right)$ satisfies $\sigma_{i}P=Z_{i}+h_{2,i}\left(pk_{2,i}^{*}+h_{1,i}^{*}P_{\mathrm{KGC}}\right).$ Therefore, it is indistinguishable from a signature produced by the original signing algorithm.

Forgery phase: eventually, $A_{2}$ outputs a purported forgery $sig_{i}^{*}=\left(Z_{i}^{*},\sigma_{i}^{*}\right)$ on $(M_{i}^{*},PID_{i}^{*},pk_{i}^{*},t_{i}^{*}).$ The forgery is valid only if $A_{2}$ has not requested the target secret value $x_{i}^{*}$ or a signature on the same message tuple.

The challenger first verifies the consistency of the complete public key by checking $pk_{2,i}^{*}\overset{?}{=}Y_{i}^{*}+h_{1,i}^{*}X_{i}^{*}.$ It then verifies $\sigma_{i}^{*}P\overset{?}{=}Z_{i}^{*}+h_{2,i}^{*}\left(pk_{2,i}^{*}+h_{1,i}^{*}P_{\mathrm{KGC}}\right)$.

By applying the Forking Lemma to the relevant $H_{2}$ query, the challenger obtains another valid forgery $\left(Z_{i}^{*},{\hat{\sigma }}_{i}^{*}\right)$ with the same $PID_{i}^{*}$, $M_{i}^{*}$, $Z_{i}^{*}$, $t_{i}^{*}$, complete public key $pk_{i}^{*}$, and $H_{1}$ output $h_{1,i}^{*}$, but with $h_{2,i}^{*}\neq {\hat{h}}_{2,i}^{*}.$ Hence, $\sigma_{i}^{*}P=Z_{i}^{*}+h_{2,i}^{*}\left(pk_{2,i}^{*}+h_{1,i}^{*}P_{\mathrm{KGC}}\right)$ and ${\hat{\sigma }}_{i}^{*}P=Z_{i}^{*}+{\hat{h}}_{2,i}^{*}\left(pk_{2,i}^{*}+h_{1,i}^{*}P_{\mathrm{KGC}}\right).$ Subtracting the above equations yields $\left(\sigma_{i}^{*}-{\hat{\sigma }}_{i}^{*}\right)P=\left(h_{2,i}^{*}-{\hat{h}}_{2,i}^{*}\right)\left(pk_{2,i}^{*}+h_{1,i}^{*}P_{\mathrm{KGC}}\right).$ Define $w_{i}=\left(\sigma_{i}^{*}-{\hat{\sigma }}_{i}^{*}\right){\left(h_{2,i}^{*}-{\hat{h}}_{2,i}^{*}\right)}^{-1}.$ According to the construction of the target public key,

(8) $$\begin{array}{cc} pk_{2,i}^{*}+h_{1,i}^{*}P_{\mathrm{KGC}} & =Y_{i}^{*}+h_{1,i}^{*}X_{i}^{*}+h_{1,i}^{*}P_{\mathrm{KGC}}\\ \; & =y_{i}^{*}P+h_{1,i}^{*}\lambda P+h_{1,i}^{*}\beta P\\ \; & =\left(y_{i}^{*}+h_{1,i}^{*}\beta +h_{1,i}^{*}\lambda \right)P\\ \; & =\left(d_{i}^{*}+h_{1,i}^{*}\lambda \right)P. \end{array}$$

It follows that $w_{i}=d_{i}^{*}+h_{1,i}^{*}\lambda .$ Therefore, the challenger extracts the ECDLP solution as $\lambda =\left(w_{i}-d_{i}^{*}\right){\left(h_{1,i}^{*}\right)}^{-1}.$

If $A_{2}$ produces a valid forgery with non-negligible probability, then, after the polynomial losses caused by guessing the target pseudonym and applying the Forking Lemma, ${𝒞}_{2}$ solves the ECDLP with non-negligible probability. This contradicts the assumed hardness of the ECDLP in G. Therefore, the proposed CL-ACPPA scheme is existentially unforgeable against adaptive chosen-message attacks launched by a Type-II adversary. □

### 4.2. Security Requirement Analysis

This section analyzes our scheme based on the security requirements for VANETs. To comprehensively evaluate the security advantages of the proposed scheme, Table 2 systematically compares CL-ACPPA with existing representative schemes across key security attributes.

(1) Message authentication and integrity. After receiving a message, the verifier checks whether the equation $\sigma_{i}\cdot P=Z_{i}+h_{2,i}\left(pk_{2,i}+h_{1,i}P_{\mathrm{KGC}}\right)$ holds. This process ensures both identity authentication and message integrity.
(2) Identity anonymity. The pseudonym $PID_{i}$ is used to hide the real identity $ID_{i}$ of the vehicle. To reveal $ID_{i}$, an attacker must compute TRA’s secret key α. However, based on the hardness of the ECDLP, no PPT attacker can obtain α. Furthermore, because the path $V_{i}\to RSU\to TRA$ is constructed during the pseudonym request phase, an attacker cannot obtain the sender’s real location information through analysis.
(3) Conditional traceability. When it is necessary to hold a vehicle accountable, TRA can compute the vehicle’s real identity $ID_{i}=PID_{i,1}⊕H_{0}(T_{i}\;\|\;EncodePoint\left(\alpha K_{i})\right)$. And the adversary cannot obtain TRA’s private key α. This traceability relies on the assumption that the pseudonym has not been tampered with; if tampered with, the calculation yields an invalid or meaningless identity.
(4) Resistance to replay attacks. Due to the use of two timestamps, $t_{i}$ and $T_{i}$, the proposed scheme is resistant to replay attacks.
(5) Resistance to injection attacks. Suppose two malicious attackers collude to generate invalid signatures $\sigma_{k}^{\prime }=\sigma_{k}+a$ and $\sigma_{k+1}^{\prime }=\sigma_{k+1}-a$ by selecting a random element $a\in Z_{q}^{*}$. Clearly, by employing SET technology in the batch verification algorithm to multiply each signature by a random small coefficient $s_{i}$, the offset condition is disrupted. This ensures that, since $s_{k}\neq s_{k+1}$, the injected message *a* cannot be cancelled by −a; thus, $s_{k}a\neq s_{k+1}a$.
  (9) $$\begin{array}{cc} \left(\sum_{i=1}^{n}s_{i}\sigma_{i}^{\prime }\right)P & =\left(\sum_{i=1}^{k-1}s_{i}\sigma_{i}+s_{k}\sigma_{k}^{\prime }+s_{k+1}\sigma_{k+1}^{\prime }+\sum_{i=k+2}^{n}s_{i}\sigma_{i}\right)P\\ \; & =\left(\sum_{i=1}^{k-1}s_{i}\sigma_{i}+s_{k}\sigma_{k}+s_{k}a+s_{k+1}\sigma_{k+1}-s_{k+1}a+\sum_{i=k+2}^{n}s_{i}\sigma_{i}\right)P\\ \; & \neq \sum_{i=1}^{n}s_{i}Z_{i}+\sum_{i=1}^{n}s_{i}h_{2,i}pk_{2,i}+\left(\sum_{i=1}^{n}s_{i}h_{2,i}h_{1,i}\right)P_{\mathrm{KGC}} \end{array}$$
(6) Resistance to signature swap attack. Without loss of generality, let n=2. Assume the attacker generates two single valid signatures, $\sigma_{1}$ and $\sigma_{2}$. The signed messages are set as $\left(Z_{1},\sigma_{2},t_{1},PID_{1},M_{1},pk_{1}\right)$ and $\left(Z_{2},\sigma_{1},t_{2},PID_{2},M_{2},pk_{2}\right)$, where the signatures $\sigma_{1}$ and $\sigma_{2}$ are swapped. Clearly, due to the SET technique in the batch verification algorithm, the small exponents $s_{1}$ and $s_{2}$ corresponding to $\sigma_{2}$ and $\sigma_{1}$ on the left side of the verification equation do not match those on the right side.
  (10) $$\begin{array}{cc} (s_{1}\sigma_{2}+s_{2}\sigma_{1})P & =\left(s_{1}Z_{2}+s_{2}Z_{1}\right)+s_{1}h_{2,2}pk_{2,2}+s_{2}h_{2,1}pk_{2,1}+(s_{1}h_{2,2}h_{1,2}\\ \; & \;+s_{2}h_{2,1}h_{1,1})P_{\mathrm{KGC}}\\ \; & \neq \sum_{i=1}^{2}s_{i}Z_{i}+\sum_{i=1}^{2}s_{i}h_{2,i}pk_{2,i}+\left(\sum_{i=1}^{2}s_{i}h_{2,i}h_{1,i}\right)P_{\mathrm{KGC}} \end{array}$$
  Therefore, the verifier will not accept these two swapped, invalid signatures.
(7) Resistance to passive eavesdropping attack. During pseudonym generation, the vehicle’s real identity is protected by ECC-based authenticated hybrid encryption. The outer encryption layer can be removed only by the intended RSU, while the inner ciphertext can be decrypted only by the TRA using its private key. Therefore, an external passive eavesdropper cannot recover the vehicle’s real identity from intercepted pseudonym generation messages, assuming the confidentiality of the underlying encryption scheme.
(8) Resistance to pseudonym tampering attack. During pseudonym generation, TRA signs the pseudonym $PID_{i}$ as $\sigma_{t}=Sig_{\alpha }\left(PID_{i}\right)$. The vehicle only accepts $PID_{i}$ after verifying: $Ver_{T_{\mathrm{TRA}}}\left(PID_{i},\sigma_{t}\right)=1$.
  Before generating the partial private key, KGC first verifies whether TRA’s signature is valid. If an external adversary or malicious intermediary attempts to replace $PID_{i}$ with a forged $PID_{i}^{\prime }$ during the transmission from $V_{i}$ to KGC, it must construct a new signature $\sigma_{t}^{\prime }$ such that $Ver_{T_{\mathrm{TRA}}}\left(PID_{i}^{\prime },\sigma_{t}^{\prime }\right)=1$. This is equivalent to forging an elliptic curve-based digital signature scheme, which contradicts the unforgeability assumption. Therefore, the adversary cannot replace the pseudonym without the vehicle’s knowledge, and the proposed scheme can effectively resist pseudonym tampering attacks.
(9) Resistance to public key replacement attacks. In the proposed scheme, the complete public key of $V_{i}$ is $pk_{i}=\left(X_{i},Y_{i},pk_{2,i}\right),$ where $X_{i}=x_{i}P,Y_{i}=y_{i}P,$ and $pk_{2,i}=Y_{i}+h_{1,i}X_{i},h_{1,i}=H_{1}(PID_{i}∥Y_{i}∥P_{\mathrm{KGC}}∥X_{i}).$ Therefore, the three values are not treated as unrelated public key components. Instead, they form a well-defined public key record generated according to the vehicle key generation algorithm. Standard elliptic curve point-format, curve-membership, and subgroup checks are applied when the public key is parsed.

The public key is cryptographically bound at two levels. First, $X_{i}$ and $Y_{i}$ are bound to $PID_{i}$ and $P_{\mathrm{KGC}}$ through $h_{1,i}$. Second, the complete public key is included in $h_{2,i}=H_{2}(M_{i}∥PID_{i}∥pk_{i}∥Z_{i}∥t_{i}).$ Consequently, all public key components are incorporated into the existing verification equation $\sigma_{i}P=Z_{i}+h_{2,i}\left(pk_{2,i}+h_{1,i}P_{\mathrm{KGC}}\right).$ Consider a Type-I adversary that replaces the vehicle-generated component $X_{i}$ with $X_{i}^{\prime }=x_{i}^{\prime }P.$ The adversary may choose and know $x_{i}^{\prime }$. However, this replacement changes $h_{1,i}^{\prime }=H_{1}(PID_{i}∥Y_{i}∥P_{\mathrm{KGC}}∥X_{i}^{\prime })$ and requires the corresponding well-formed component $pk_{2,i}^{\prime }=Y_{i}+h_{1,i}^{\prime }X_{i}^{\prime }.$ The complete public key supplied to $H_{2}$ is therefore also changed.

To generate a valid signature under the replaced public key, the adversary requires both $x_{i}^{\prime }$ and the corresponding partial private key contribution $d_{i}^{\prime }=y_{i}+h_{1,i}^{\prime }\beta .$ Although the Type-I adversary may know $x_{i}^{\prime }$, it does not know the KGC master secret β and cannot obtain the target identity’s partial private key. Hence, it cannot compute a valid signature satisfying $\sigma_{i}^{\prime }P=Z_{i}^{\prime }+h_{2,i}^{\prime }\left(pk_{2,i}^{\prime }+h_{1,i}^{\prime }P_{\mathrm{KGC}}\right)$ except with negligible probability. As formally established in Theorem 1, a successful forgery in this case would yield a solution to the ECDLP. Therefore, the proposed scheme is resistant to Type-I public key replacement attacks.

## 5. Performance Analysis

### 5.1. Computational Efficiency

This section mainly compares the time-consuming operations. For the bilinear pairing operation, we instantiate it as follows: $e:G_{1}\times G_{1}\to G_{T}$, where $G_{1}$ is an additive group of prime order *q* on the curve $E:y^{2}=\left(x^{3}+x\right)\;\mathrm{mod}\;p$. Here, *q* and *p* are 160-bit and 512-bit prime numbers, respectively. The time consumed by a bilinear pairing operation is denoted as $T_{bp}$, and the scalar multiplication on the curve is denoted as $T_{bm}$. For operations on the elliptic curve, we instantiate it as an additive group *G* of prime order *q* on the curve $E:y^{2}=\left(x^{3}+ax+b\right)\;\mathrm{mod}\;p$, where $a,b\in Z_{q}^{*}$, and p,q are 160-bit prime numbers.

Additionally, $T_{a}$ denotes the time required to perform a point addition operation on this elliptic curve, $T_{m}$ denotes the time for a scalar multiplication operation, and $T_{h}$ denotes the time for a Map-to-Point hash function operation. In this paper, under the test environment of Windows operating system and Intel Core i5-1035G1 CPU @ 1.00GHz processor, we use the Java Pairing-Based Cryptography library (JPBC) to take the average execution time of 10,000 times for the involved cryptographic operations as the test data. The test data are shown in Table 3.

The computational efficiency of the proposed CL-ACPPA scheme is evaluated against representative existing schemes, with particular attention to signature generation, individual signature verification, and batch verification. In the scheme proposed by Mei et al. [18], signature generation requires four scalar multiplications in $G_{1}$, two elliptic curve point additions, and two Map-to-Point hash operations, resulting in a computational cost of $4T_{bm}+2T_{a}+2T_{h}\approx 37.4152\;\mathrm{ms}.$ The corresponding individual signature verification cost is $4T_{bp}+2T_{bm}+2T_{h}\approx 41.4730\;\mathrm{ms},$ while the batch verification cost for *n* signatures is $4T_{bp}+2nT_{bm}+2T_{h}+\left(2n-2\right)T_{a}\approx \left(14.3102n+27.1628\right)\;\mathrm{ms}.$

For the proposed CL-ACPPA scheme, the SET-based aggregation performed by the RSU incurs a cost of $nT_{sm}+\left(n-1\right)T_{a},$ whereas the subsequent AS-side batch verification incurs $\left(n+2\right)T_{m}+\left(n+1\right)T_{a}.$ Therefore, the total aggregation and verification cost is $nT_{sm}+\left(n+2\right)T_{m}+2nT_{a}=\left(2.9042n+3.8412\right)\;\mathrm{ms}.$

**Figure 3:**
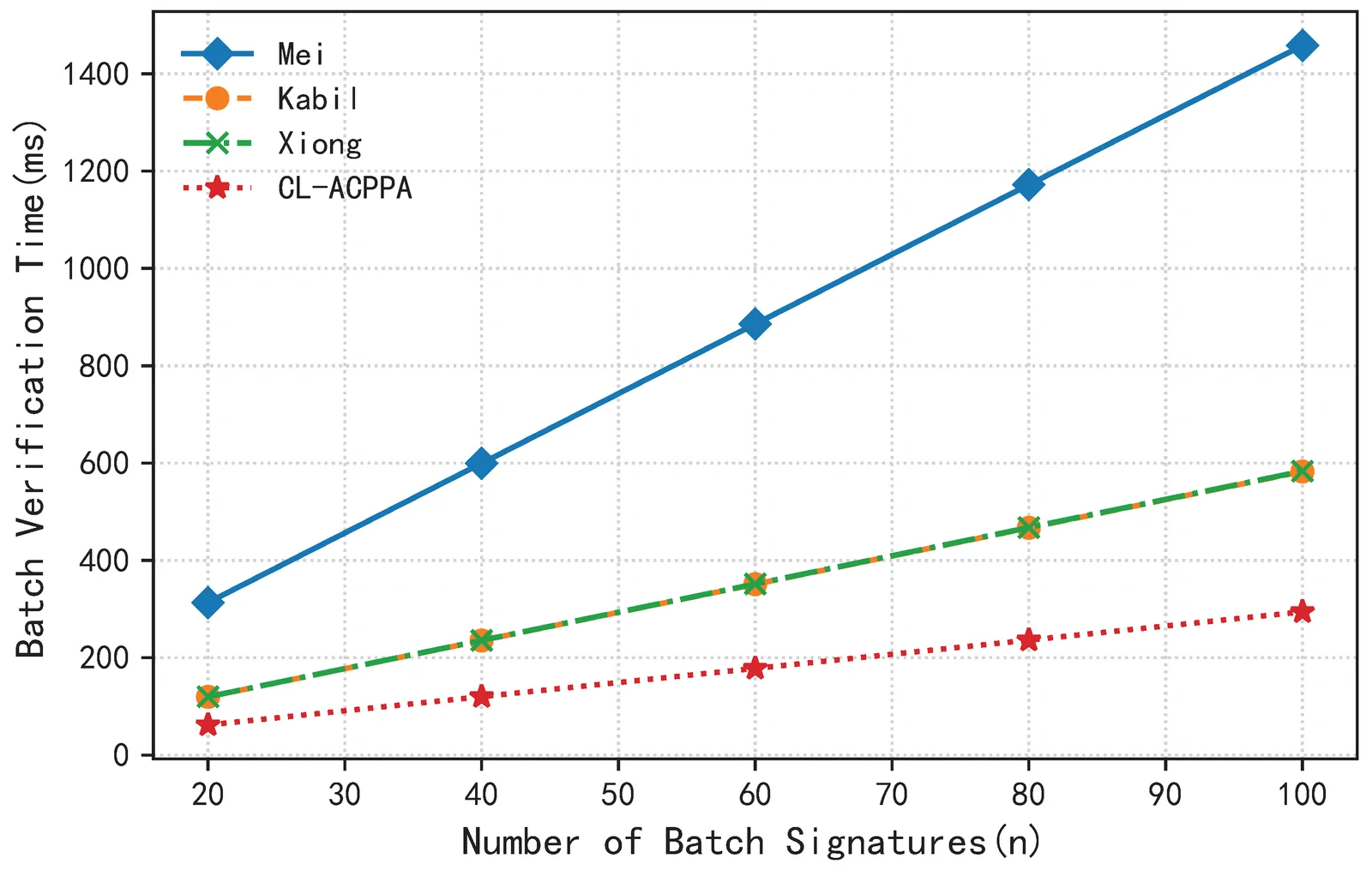
Relationship between computational cost and number of signatures.

This expression explicitly accounts for the small-scalar multiplications introduced by the SET mechanism and therefore reflects the complete computational cost of aggregation and batch verification. Compared with the scheme of Mei et al. [18], CL-ACPPA reduces the signature generation and individual signature verification costs by 94.87% and 86.07%, respectively. For n=100, CL-ACPPA requires 294.2612ms, corresponding to reductions of 79.82% and 49.57% relative to the schemes of Mei et al. [18] and Xiong et al. [25], respectively. The computational costs of the Kabil, Xiong, and Xu schemes are summarized in Table 4. As illustrated in Figure 3, the overall computational cost of CL-ACPPA remains lower than those of the Mei, Kabil, and Xiong schemes as the batch size increases. Although SET-based aggregation introduces additional small-scalar multiplications compared with unweighted aggregation, these operations strengthen the aggregation process by preventing the algebraic cancellation exploited in injection and signature-swapping attacks. Consequently, the proposed scheme achieves a favorable balance between computational efficiency and resistance to these attacks.

### 5.2. Required Signature Length and Communication Overhead

In this analysis, we consider that an element in group *G* is 40 bytes, an element in $G_{1}$ is 128 bytes, an element in $Z_{q}^{*}$ is 20 bytes, and a timestamp is allocated 4 bytes, as shown in Table 5.

Table 6 reports the recurring communication overhead associated with vehicle-generated authentication messages. The analysis includes the pseudonym, timestamps, signature, and public key components transmitted with each message, while excluding the application-message payload, global system parameters, and long-term authority public keys distributed during initialization or registration. Communication between the RSU and the AS is also excluded. This accounting boundary is applied uniformly to all compared schemes, thereby ensuring a consistent and equitable comparison.

For CL-ACPPA, the communication overhead of a single authentication message is 208 bytes. Accordingly, the communication overhead for transmitting n authentication messages is 208n bytes. The SET coefficients are generated locally by the RSU after receiving the authentication messages and therefore do not contribute to the vehicle-side communication overhead. Compared with the Mei scheme, CL-ACPPA reduces the single message communication overhead by 62.59%. Compared with the Xiong and Xu schemes, it incurs an additional 40 bytes per message because its complete public key contains one additional elliptic curve point. This moderate increase supports the proposed public key structure and its associated security properties. As indicated in Table 2, the compared schemes do not provide the same combination of security guarantees as CL-ACPPA: the Mei, Xiong, and Kabil schemes lack resistance to passive eavesdropping, whereas none of the compared schemes provides protection against pseudonym tampering.

### 5.3. Algorithmic Comparison with Xiong et al. [25]

Although both Xiong et al.’s scheme and the proposed CL-ACPPA scheme are constructed using ECC and avoid bilinear pairing operations, their aggregation mechanisms are different. In their scheme, an individual signature is computed as $T_{i}=\left(psk_{i}+h_{i}vsk_{i}\right)\;\mathrm{mod}\;q,$ and the aggregate signature component is obtained through the unweighted summation $T=\sum_{i=1}^{n}T_{i}.$ The principal batch verification relation is consequently based on the following unweighted linear equation:

(11) $$TP=\sum_{i=1}^{n}A_{i}+\sum_{i=1}^{n}Q_{i}P_{\mathrm{KGC}}+\sum_{i=1}^{n}h_{i}vpk_{i}$$

To strengthen the aggregation process, Xiong et al. additionally select $agg_{j}\in Z_{q}^{*}$, compute $agg_{\mathrm{KGC},j}=agg_{j}P$, and derive $\beta_{j}=H_{3}\left(T_{1}agg_{\mathrm{KGC},j}\;∥\;T_{2}agg_{\mathrm{KGC},j}\;∥\;\cdots \;∥\;T_{n}agg_{\mathrm{KGC},j}\right).$ Because every $T_{i}$ is a full-length element of $Z_{q}^{*}$, constructing the *n* elliptic curve points $T_{i}agg_{\mathrm{KGC},j}$ requires *n* general scalar multiplications. Thus, Xiong et al.’s construction relies on an additional hash-based binding value outside the principal unweighted aggregate equation and introduces extra full-length scalar-multiplication operations. In contrast, CL-ACPPA applies the SET coefficients directly to the individual signature components: $\sigma =\sum_{i=1}^{n}s_{i}\sigma_{i},Z=\sum_{i=1}^{n}s_{i}Z_{i}.$ Accordingly, the coefficients also appear directly in the batch verification equation:

(12) $$\sigma P=Z+\sum_{i=1}^{n}s_{i}h_{2,i}\left(pk_{2,i}+h_{1,i}P_{\mathrm{KGC}}\right)$$

As formally analyzed in Section 4.2 (5)(6), the SET coefficients directly disrupt the algebraic cancellation condition exploited by injection and signature-swapping attacks, and an invalid batch passes verification with probability at most $2^{-\ell }$.

## 6. Discussion

### 6.1. Communication Overhead Scope and Deployment Considerations

The communication comparison in Table 6 focuses on the recurring vehicle-side communication overhead incurred when authentication messages are transmitted to the RSU. It includes the pseudonym, timestamp, signature, and any public key components that must accompany an authentication message. Global system parameters and long-term authority public keys distributed during initialization or registration are not included because they can be authenticated, cached, and reused. The same accounting boundary is applied to all compared schemes to ensure a consistent and like-for-like comparison.

Nevertheless, these parameters introduce additional bootstrap and maintenance traffic in practical deployment, particularly when a vehicle enters a new administrative domain, clears its cache, receives updated parameters, or processes key-renewal and revocation information. If a verifier has not cached the sender’s public key or if the public key is changed frequently, the corresponding public key transmission and authentication overhead must also be considered. Consequently, Table 6 should be interpreted as an online authentication message comparison rather than a complete measurement of all lifecycle communication costs. Future field deployment should additionally evaluate initialization, public key acquisition, revocation and update information, and retransmissions caused by unreliable wireless links.

### 6.2. Limitations of Conditional Traceability

The TRA signature and the mandatory verification performed by the KGC prevent a pseudonym modified during transmission from being used to obtain a valid partial private key unless the adversary can forge the TRA’s signature. Nevertheless, conditional traceability is not an unconditional guarantee. The identity recovery operation $ID_{i}=PID_{i,1}⊕H_{0}\left(T_{i}\;\|\;\mathrm{EncodePoint}\left(\alpha K_{i}\right)\right)$ requires the TRA private key α and the authentic registration information associated with the pseudonym. Therefore, traceability relies on the security and availability of the TRA private key and the integrity and availability of the required registration records.

Traceability may fail or become temporarily unavailable if the TRA private key or registration records are lost, corrupted, or unavailable; if historical information is deleted after key rotation; if enrollment information is incorrect; or if the TRA and its database are affected by denial-of-service attacks. A compromised vehicle device may also allow credential cloning, while a compromised or malicious TRA may perform unauthorized identity disclosure or create an incorrect association between a pseudonym and a real identity. The latter case is outside the trusted-TRA model considered in this work. Secure key backup and rotation, authenticated and redundant registration records, integrity-protected audit logs, historical-key retention, and timely credential revocation can mitigate these operational risks. Threshold-based or multi-authority tracing may be investigated in future work to reduce reliance on a single TRA. Accordingly, the conditional traceability claim holds under the assumptions that the TRA behaves honestly, its private key remains secure and available, and the registration information required for identity recovery remains authentic and accessible.

## 7. Conclusions

This paper points out that existing certificateless conditional privacy-preserving authentication schemes struggle to simultaneously meet security and practical deployment requirements under weak channel models. Specifically, taking the scheme in reference [26] as an example, cryptographic analysis indicates that currently proposed schemes face security risks of pseudonym tampering attacks during the pseudonym generation and transmission stages, revealing deficiencies in pseudonym integrity protection and end-to-end verifiability. To address this issue, we propose a CL-ACPPA scheme, which enhances security by introducing a verifiable pseudonym binding mechanism. The security of our scheme is proven to be unforgeable under the ROM, relying on the hardness assumption of ECDLP. Through security and performance analyses, the CL-ACPPA scheme not only fulfills security requirements such as user anonymity and conditional traceability, but also resists injection attacks and signature-swapping attacks initiated by malicious vehicles during the aggregation process, while further improving performance. Future research will explore integrating emerging technologies into vehicular network security authentication mechanisms to continuously enhance the overall trustworthiness and communication security of systems in complex network environments.

## Figures and Tables

**Table 1:** Symbol description and definitions.

Notation	Description
β	Private key of the KGC
$T_{i}$	Validity period
$P_{\mathrm{KGC}}$	Public key of the KGC
$t_{i}$	Timestamp
α	Private key of the TRA
$d_{i}$	Partial private key of vehicle $V_{i}$
$T_{TRA}$	Public key of the TRA
$sk_{i}$	Full private key of vehicle $V_{i}$
$r_{i}$	Private key of the *i*-th RSU
$pk_{i}$	Public key of vehicle $V_{i}$
$R_{i}$	Public key of the *i*-th RSU
$sig_{i}$	Signature of the message
$V_{i}$	The *i*-th vehicle

**Table 2:** Security comparison of different schemes.

Scheme	Pairing-Free	Type-I Attack	Type-II Attack	Injection & Sig. Swap	Passive Eavesdropping	Conditional Traceability	Security
Mei [18]	×	✓	✓	✓	×	✓	×
Kabil [26]	✓	✓	✓	✓	×	×	×
Xiong [25]	✓	✓	×	✓	×	×	×
Xu [24]	✓	✓	✓	×	✓	×	×
CL-ACPPA	✓	✓	✓	✓	✓	✓	✓

Note: ✓ and × indicate that the corresponding security attribute is provided or not provided, respectively. “Sig.” denotes signature, and CL-ACPPA represents the proposed scheme.

**Table 3:** Symbols and running time.

Symbol	Description	Time (ms)
$T_{a}$	Point addition on elliptic curve	0.0086
$T_{m}$	Scalar multiplication kP, P∈G	1.9206
$T_{bm}$	Scalar multiplication kP, $P\in G_{1}$	7.1465
$T_{h}$	Map-to-Point hash operation	4.4060
$T_{bp}$	Bilinear pairing e(P,Q), $P,Q\in G_{1}$	4.5920
$T_{sm}$	Small-scalar multiplication sP, $s\in [1,2^{\ell }]$, ℓ=80	0.9664

**Table 4:** Computational efficiency comparison.

Scheme	Signing Time (ms)	Single Signature Verification Time (ms)	Batch Verification Time (ms)
Mei [18]	$4T_{bm}+2T_{a}+2T_{h}\approx 37.4152$	$4T_{bp}+2T_{bm}+2T_{h}\approx 41.4730$	$4T_{bp}+2nT_{bm}+2T_{h}+\left(2n-2\right)T_{a}\approx 14.3102n+27.1628$
Kabil [26]	$T_{m}\approx 1.9206$	$3T_{m}+3T_{a}\approx 5.7876$	$\left(3n+2\right)T_{m}+3nT_{a}\approx 5.7876n+3.8412$
Xiong [25]	$T_{m}\approx 1.9206$	$3T_{m}+2T_{a}\approx 5.7790$	$\left(3n+2\right)T_{m}+4nT_{a}\approx 5.7962n+3.8412$
Xu [24]	$T_{m}\approx 1.9206$	$3T_{m}+2T_{a}\approx 5.7790$	$\left(n+2\right)T_{m}+\left(2n+1\right)T_{a}\approx 1.9378n+3.8498$
CL-ACPPA	$T_{m}\approx 1.9206$	$3T_{m}+2T_{a}\approx 5.7790$	$nT_{sm}+\left(n+2\right)T_{m}+2nT_{a}\approx 2.9042n+3.8412$

**Table 5:** Sizes of different element types in communication.

Element Type	Description	Size (Bytes)
$|G_{1}|$	Group element in $G_{1}$	128
|G|	Group element based in *G*	40
$|Z_{q}^{*}|$	Element in finite field	20
$|t_{i}|$	Timestamp	4

**Table 6:** Comparison of communication costs.

Scheme	Signature Length (Bytes)	Single Message Transmission Costs (Bytes)	Batch Transmission Costs (Bytes)
Mei [18]	$2|G_{1}|=256$	$4|G_{1}|+2\left|Z_{q}^{*}\right|+\left|t_{i}\right|=556$	556n
Kabil [26]	$\left|G\right|+|Z_{q}^{*}|=60$	$4\left|G\right|+2|Z_{q}^{*}|+2|t_{i}|=208$	208n
Xiong [25]	$\left|Z_{q}^{*}\right|=20$	$3|G|+2\left|Z_{q}^{*}\right|+2\left|t_{i}\right|=168$	168n
Xu [24]	$\left|G\right|+\left|Z_{q}^{*}\right|=60$	$3|G|+2\left|Z_{q}^{*}\right|+2\left|t_{i}\right|=168$	168n
CL-ACPPA	$\left|G\right|+\left|Z_{q}^{*}\right|=60$	$4|G|+2\left|Z_{q}^{*}\right|+2\left|t_{i}\right|=208$	208n

## Data Availability

Data supporting the reported results can be obtained from the corresponding author upon reasonable request.
